# Supplementary material for: Speed-up and slow-down of a quantum particle
Source: Sci Rep. 2022 Mar 9;12:3842. doi: 10.1038/s41598-022-07599-1 (PMC8907271; doi:10.1038/s41598-022-07599-1)
Supplement: Supplementary file 1 — Supplementary Information. [file 41598_2022_7599_MOESM1_ESM.pdf]

Supplementary Information

I. APPENDIX A. FREELY PROPAGATING ENVELOPES AND THE ERROR FUNCTIONS IN EQ.(49)

A freely propagating Gaussian envelope $G_0(x,t)$ in equation (8) has the form

$$G_0(x,t) = \left(\frac{2\Delta_x^2}{\pi\Delta_{x_t}^4}\right)^{1/4} \exp\left[-(x-p_0t/\mu-x_0)^2/\Delta_{x_t}^2\right] \tag{S1}$$

where $\Delta_{x_t} = \sqrt{\Delta x^2 + 2it/\mu}$. The integrals in equation (18) can be expressed in terms of the error function [1], and we have

$$\Psi^T(x,t) = \exp\left[ip_0x - iE(p_0)t\right]\{G_0(x,t) + \sum_{n_B}\mathrm{Res}(k_{n_B})\mathfrak{G}^B(x,k_n^I,p_0) + \sum_{n_R}\mathrm{Res}(k_{n_R})\mathfrak{G}^R(x,k_n^I,p_0)\} \tag{S2}$$

where

$$\mathfrak{G}^{B,R} = \left(\frac{2\Delta_x^2}{\pi\Delta_{x_t}^4}\right)^{1/4} \times \frac{\sqrt{\pi}\left[1\pm\mathrm{erf}\left(b_{B,R}/2\sqrt{a}\right)\right]}{2\sqrt{a}}\exp[b_{B,R}^2/4a + c] \tag{S3}$$

and

$$\begin{aligned} &a = -1/\Delta_{x_t}^2, \quad c = -(x-pt-x_0)^2/\Delta_{x_t}^2, \\ &b_{B,R} = i(k_{n_{B,R}} - p_0) + 2(x-pt-x_0)/\Delta_{x_t}^2. \end{aligned} \tag{S4}$$

For an Eckart well with $s = M$, we have equation (49).

II. APPENDIX B. BEHAVIOUR OF $\tilde{\eta}(p_0, x')$ FOR $x' \to 0$

To see whether $\tilde{\eta}(p_0, x')$ remains finite at $x' = 0$, we note that using (6) one can write the integral in equation (20) as

$$I \equiv \int_{-\infty}^{\infty}[T(k,V)-1]dk = \int_0^{\infty}\{\mathrm{Re}[T(k,V)]-1\}dk. \tag{S5}$$

Since $|T(k,V)| \le 0$, $\tilde{\eta}(p_0, x=0)$ can only become infinite due to the behaviour of the integrand at large k. As $k\to\infty$ the motion becomes semiclassical, and from (10) we have

$$I = \int_0^{\infty}\{\cos\left[\Phi(k,V)\right]-1\}dk, \tag{S6}$$

where for $\Phi(k,V)-1$ we find

$$\Phi(k\to\infty,V) - 1 \to (\mu/k)\int_{-\infty}^{\infty}V(x)dx + o(1/k). \tag{S7}$$

With $J \equiv \int_{-\infty}^{\infty} V(x)dx$ finite, we have

$$\cos[\Phi(k\to\infty,V)] - 1 \to -\mu^2J^2/2k^2, \tag{S8}$$

integral (S6) converges to a finite value, and we find $\tilde{\eta}(p_0,x') < \infty$. This is true for both barriers, $U_0 > 0$, and wells, $U_0 < 0$.

III. APPENDIX C. DOUBLE POLES OF THE TRANSMISSION AMPLITUDE $T(p,V)$

In a special case $s = M+1/2$, $M = 0, 1, ...$ on the imaginary axis there are $2M+2$ simple poles k_n^I, $n = 0, 1, ..., 2M+1$, $M+1$ above, and $M+1$ below the real axis (cf. Fig. 3). The rest are double poles, since both Gamma functions in the numerator of equation (4) diverge at $p = k_n^I$, $n \geq 2M+2$. The corresponding residues can be obtained with the help of the Cauchy's differentiation formula

$$\mathrm{Res}_2(k_n^I) = \frac{2i\alpha}{n!(n+2M+2)!} \times \left(\sum_{k=1}^{k=n} \frac{1}{k} + \sum_{k=n+1}^{k=n+2M+2} \frac{1}{2k} - \gamma(1) \right) \times \frac{1}{\Gamma(-n-M-3/2)\Gamma(-n-M-1/2)}, \tag{S9}$$

where $\gamma(1) \approx 0.5772$ is the Euler-Mascheroni constant [1]. The final result, therefore, is

$$\eta(p_0, x') = \delta(x') + i\exp(-ip_0x') \times \begin{cases} \sum_{n=0}^{M} Res(k_n^I)\exp(ik_n^I x'), & x' \geq 0 \\ \left[\sum_{n=M+1}^{2M+1} Res(k_n^I)\exp(ik_n^I x') + \right. \\ \left. \sum_{n=2M+2}^{\infty} Res_2(k_n^I)\exp(ik_n^I x') \right], & x' < 0. \end{cases} \tag{S10}$$

IV. APPENDIX D. THE RESIDUES IN THE LIMIT $n \to \infty$

For a large $n \to \infty$ we have

$$\Gamma(z) \approx z^{z-1/2} e^{-z} \sqrt{2\pi}, \tag{S11}$$

which is valid for $|\arg(z)| < \pi$. Using the Sterling formula $n! \approx \sqrt{2\pi n} \left(\frac{n}{e}\right)^n$ and applying this to the residues of the poles of the first kind in equation (30), yields

$$\mathrm{Res}(k_n^I) \approx i \frac{\alpha(-1)^n}{2\pi\sqrt{n}} \times \frac{(-n+s)^{n-s+1/2}(-n+s+1)^{n-s-1/2}}{n^n(n-2s-1)^{-n+2s-1}}. \tag{S12}$$

where

$$\lim_{n\to\infty} \frac{(-n+s)^{n-s+1/2}(-n+s+1)^{n-s-1/2}}{(n-2s-1)^{-n+2s-1} n^n} = i(-1)^n\sqrt{n}. \tag{S13}$$

Recalling that $\mathrm{Res}(k_n^I) = -\mathrm{Res}^*(k_n^{II})$, we have the desired limit

$$\lim_{n\to\infty} \mathrm{Res}(k_n^I) = -\alpha/2\pi, \quad \lim_{n\to\infty} \mathrm{Res}(k_n^{II}) = \alpha/2\pi. \tag{S14}$$

V. APPENDIX E. THE DOUBLE-SLIT ANALOGY

To see what the double-slit conundrum and the problem at hand have in common, consider the simplest double-slit arrangement. This includes a two-level system with a Hamiltonian $\hat{H}_S$, prepared at $t = 0$ is a state $|\psi\rangle$ (the source), and observed again in a final state $|\phi\rangle$ (point on the screen). A pair of orthogonal state $|b_1\rangle$ and $|b_2\rangle$ in which the system could be at $t = T/2$ play the role of the "slits", so that the system can reach the $|\phi\rangle$ by passing via $|b_1\rangle$ or $|b_2\rangle$, the corresponding amplitudes being $[\hat{U}_S(t) = \exp(-i\hat{H}_S)]$

$$A(\phi \leftarrow b_n \leftarrow \psi) = \langle\phi|\hat{U}_S(T/2)|b_n\rangle\langle b_n|\hat{U}_S(T/2)|\psi\rangle \equiv \eta_n, \quad n = 1, 2. \tag{S19}$$

The amplitude to arrive in $|\phi\rangle$ results from the interference between the two alternatives, and the corresponding probability is $|\eta_1 + \eta_2|^2$. The Uncertainty Principle (UP) [2] states that this "which way?" question *cannot be answered without destroying the interference.* In order to determine the route taken by the system we can measure, at $t = T/2$, the "slit number operaror"

$$\hat{\mathcal{N}}_S = 1 \times |b_1\rangle\langle b_1| + 2 \times |b_2\rangle\langle b_2|, \tag{S20}$$

thus obtaining the result $n = 1, 2$, if the n-th route is taken. To do so we couple the system to a von Neumann pointer with position x, so that the full Hamiltonian becomes $\hat{H} = \hat{H}_S - i\partial_x \hat{\mathcal{N}}_S$, and the initial state of the joint system is $|\psi\rangle \otimes |G\rangle$, where the pointer's initial state can be chosen to be a real-valued Gaussian of a width Δx, centred at the origin, $\langle x|G\rangle = \langle -x|G\rangle$. Now the (unnormalised) probability to find the pointer at x, given that the system has arrived at $|\phi\rangle$ is

$$\rho(x) = |G(x-1)\eta_1 + G(x-2)\eta_2|^2, \tag{S21}$$

and everything depends on the accuracy of the measurement, Δx. If $\Delta x << 1$, each trial produces an outcome 1 or 2, we know where the system was at $t = T/2$, but the probability to arrive in $|\phi\rangle$ has changed to $|\eta_1|^2 + |\eta_2|^2$. We destroyed the studied transition.
To keep the transition more or less intact, we can try choosing a large Δx, $\Delta x \to \infty$. Now the probability of post-selection in $|\phi\rangle$, and the pointer's readings may lie anywhere, $-\infty < x < \infty$. This agrees with the UP, which says that the route taken by the system cannot be determined in the presence of interference. We can, however, evaluate the *mean* pointer position which is easily found to be [cf. equation (10)]

$$\langle x\rangle \equiv \int x\rho(x)dx / \int \rho(x)dx \approx \mathrm{Re}\left[\frac{1\times\eta_1 + 2\times\eta_2}{\eta_1+\eta_2}\right] \quad , \tag{S22}$$

and treat $\langle x\rangle$ as the mean slit number, $\overline{n}$, in the presence of interference. The problem is that with complex values η_n, with no restrictions on the sign of either $\mathrm{Re}[\eta_n]$ and $\mathrm{Im}[\eta_n]$ there are also no restrictions on the value of $\langle x\rangle$. For example [7] it is easy to find $|\psi\rangle$, $|\phi\rangle$, $|b_1\rangle$ and $|b_2\rangle$ for our mean slit number to be 100.
Can we measure $\overline{n} = \langle x\rangle = 100$? Definitely yes.
Do we really want to "explain" a situation, where we drilled only two holes in the screen, by saying that there are up to 100 holes we did not know about? Most likely not.
The same applies to the phase time τ_{phase} in equation (47), where the particle's own position x plays the role of the pointer's coordinate in (S21) [4]. As the width of the wave packet becomes very large, $\Delta x \to \infty$, for the position of the COM we have

$$x_{COM} = \langle x\rangle \equiv \int x|\psi(x,T)|^2 dx / \int |\psi(x,T)|^2 dx \approx \mathrm{Re}\left[\frac{\int x'\eta(x')dx'}{\int \eta(x')dx'}\right] \tag{S23}$$

When this is used to deduce the value of τ_{phase}, the value turns out to be very small.
Can we measure this short duration? Definitely yes [5].
Do we really want to claim that a tunnelling particle defies relativity by moving too fast in the barrier, when all the barrier can do is delay it? Most likely not (with few exceptions [6]).

So what is the meaning of the (measured) values (S22) and (S23)? They express the correct relations between Feynman's transition amplitude [2], which quantum mechanics uses to describe the phenomena in question, and very little else [7].

VI. APPENDIX F. THE CENTRE-OF-MASS DELAY FOR $s \approx M$, $M = 1, 2, 3...$

For $s \approx M$, where the M-th bound state enters the well as s increases, we have

$$\begin{aligned}\tilde{\eta}(p,x') \approx \delta(x') &+ \sum_{n=0}^{M-1} i\,\frac{\alpha(-1)^n(2M-n)!}{n!(M-n-1)!(M-n)!} \times \exp\{-[\alpha(M-n)+ip]\,x'\}\theta(x') \\ &+ i\frac{\alpha(-1)^M(2M-1)!}{M!(M-1)!}(s-M)\exp\{-[\alpha(s-M)+ip]\,x'\} \times [\theta(x')\theta(s-M)+\theta(-x')\theta(M-s)],\end{aligned} \tag{S15}$$

and

$$T(p,V) \approx \exp[i\Theta(p)] - \frac{(-1)^M(2M-1)!}{M!(M-1)!} \times \frac{(s-M)}{(s-M)+ip/\alpha}, \tag{S16}$$

where

$$\Theta(p) = -i\ln\left\{1 - \sum_{n=0}^{M-1}\frac{(-1)^n(2M-n)!}{n!(M-n-1)!(M-n)!} \times \frac{1}{(M-n)+ip/\alpha}\right\}. \tag{S17}$$

The centre-of-mass delay, corrected for momentum filtering, is, therefore, given by

$$\delta x^T_{COM}(t) - \delta v_0 t \approx \frac{\alpha(s-M)}{\alpha^2(s-M)^2 + p_0^2} - \partial_p \Theta(p_0). \tag{S18}$$

REFERENCES

[1] Abramovitz, M. & Stegun, I. *Handbook of mathematical functions* (National Bureau of Standards, 1972).
[2] Feynman, R. P., Leighton, R. & Sands, M. *The Feynman Lectures on Physics III* (Dover Publications, Inc., New York, 1989).
[3] Sokolovski, D., Weak measurements measure probability amplitudes (and very little else). *Phys. Lett. A,* **380**, 1593 (2016).
[4] Sokolovski, D. & Akhmatskaya, E. "Superluminal paradox" in wave packet propagation and its quantum mechanical resolution.
[5] Stenner, M.D., Gauthier, D.J. & Neifeld, M.A. The speed of information in a 'fast-light' optical medium. *Nature London* **425**, 695 (2003).
[6] Nimtz, G. Tunneling confronts special relativity. *Found. Phys.* **41**, 1193 (2011).
[7] Sokolovski, D., Weak measurements measure probability amplitudes (and very little else). *Phys. Lett. A,* **380**, 1593 (2016)
